# Explore the influence of BMI in the optimal time of weaning from sequential mechanical ventilation for severity chronic obstructive pulmonary disease

**DOI:** 10.1186/1471-227X-13-S1-S1

**Published:** 2013-07-04

**Authors:** Sun Li-dong, Guo Chang-sheng, Zhao Zi-yu

**Affiliations:** 1 Emergency Department of PLA NO.155 Central Hospital, Kaifeng 475003

## Abstract

**Objective:**

To study the clinical effect of body mass index (BMI) in the optimal time of weaning from sequential invasive-noninvasive mechanical ventilation (MV) by treating severity chronic obstructive pulmonary disease (COPD) patients.

**Methods:**

94 patients with severity COPD were divided into the control group (BMI<21) and the study group (BMI>21). These two groups were treated by similar symptomatic therapies such as mechanical ventilation, antibacterial, antispasmodic, relieving asthma, antitussive, expectorant, correction of electrolyte imbalance and acid-base balance disorders, strengthen nutritional support, etc.

**Results:**

Compared with the control group, the study group had shorter duration of invasive mechanical ventilation, non-invasive mechanical ventilation time, total mechanical ventilation time, total hospital stay (P<0.01). There are significant differences between these two groups in re-intubation rate, VAP occurred in the number of case, hospital mortality rate in 28 days (P<0.05).

**Conclusions:**

It is difficult to wean successfully from sequential mechanical ventilation for severity COPD patients (BMI<21), so BMI as one of important reference index can be used to estimate the optimal time for weaning from sequential mechanical ventilation for severity COPD patients.

## 

The traditional invasive mechanical ventilation is used to treat severity COPD patients combined with more secretion, and transfer from invasive to non invasive mechanical ventilation at the time of pulmonary infection control window (PIC Window) appeared[1], although we had become consensus on invasive-noninvasive mechanical ventilation technique in treatment of chronic obstructive pulmonary disease at acute exacerbation phase (AECOPD). There are no objective indicators to measure when weaning from invasive-noninvasive mechanical ventilation. We obtained very good effects on the optimal time of weaning from sequential invasive-non invasive mechanical ventilation by BMI recently[2].

## Methods

### Design and patients

Ninety-four AECOPD patients admitted into the intensive care unit (ICU) of our hospital from June 2008 to March 2012 were included, all of whom in accordance with the criteria of COPD guideline constituted by Chinese Society of Respiratory Diseases in 2007 and the diagnosis standard of pulmonary encephalopathy[2,3]. They were divided into the control group (BMI<21) and the study group (BMI>21) in accordance with the criteria of COPD guideline constituted by American and European Society of Respiratory Diseases in 2004, BMI<21 was underweight and should be treated by nutritional support[4].

The study group consist of 23 males and 15 females aged from 41 to 85 years old (average of 64.50±4.75 years) and the course of their diseases are from 3 months to 22.23 years (average of 62.34±3.50 months), among whom there are 6 patients with type III and 32 patients with type IV COPD. The control group consist of 32 males and 24 females aged from 42 to 86 years old (average of 63.70±3.90 years) and the course of their diseases are from 4 months to 21.70 years (average of 63.18±4.22 months), among whom there are 12 patients with type III and 44 patients with type IV COPD. There is no significant difference between these two groups in age, gender, course of diseases, type of COPD, the score of APACHE II, result of blood gas analysis (P>0.05).

### Treatment

These two groups were treated by similar symptomatic therapies such as antibacterial, antispasmodic, relieving asthma, antitussive, expectorant, correction of electrolyte imbalance and acid-base balance disorders, strengthen nutritional support, etc. Patients were ventilated using assistance controlled mechanical ventilation (ACMV) during the initial mechanical ventilation, and switched to synchronous intermittent mechanical ventilation (SIMV) and pressure support ventilation (PSV) and positive expiratory end pressure (PEEP) with the improvement of patients’ condition. Mode and parameters of mechanical ventilation were adjusted according to patients’ condition. Patients were ventilated using SIMV and PSV and PEEP, and switched to PSV and PEEP after the PIC window appeared. The level of PSV was decreased gradually to 5-8cmH_2_O with the improvement of patients’ condition; at least 12 hours later, exudation was conducted and followed by non-invasive mechanical ventilation. The non-invasive mechanical ventilation that we used were bi-level positive airway pressure-spontaneous and timing mode (BiPAP-S/T); the level of inspiration positive airway pressure (IPAP) was 4-7cmH_2_O (1cmH_2_O = 0.0198KPa), and then weaning from mechanical ventilation after spontaneous breathe smoothly, instead of nasal breathing. These two groups weighed every day and kept the balance of body fluid. The treatment of the study group was the same as that of the control group until BMI >21, then weaning from mechanical ventilation.

### Observation items

The following indices were recorded at different times including before mechanical ventilation such as BMI, variation of blood gas analysis, invasive mechanical ventilation time, non-invasive mechanical ventilation time, total mechanical ventilation time, VAP occurred rate, re-intubation rate, hospital mortality rate in 28 days.

### Statistical analysis

All parameters were expressed as the mean ±standard deviation (SD), with the use of SPSS13.0 software. All indices were analyzed by independent-sample t test and x^2^ test between the two groups, and by paired-sample t test between different times for each group. A P value less than 0.05 was considered statistically significant.

## Results

Compared with control group, the study group had shorter duration of invasive mechanical ventilation, non-invasive mechanical ventilation time, total mechanical ventilation time, total hospital stay (P<0.01). There are significant differences between these two groups in re-intubation rate, VAP occurred in the number of case, hospital mortality rate in 28 days (P<0.05). Three in the study group died because of exacerbation of respiratory muscle fatigue and no improvement of patients’ condition by the non-invasive mechanical ventilation. Of the three cases, two died of MODS, and one died of discontinuation treatment. Eleven in the control group died because of MODS. Of the eleven cases, five died of refusing endotracheal intubation, and two died of discontinuation treatment. 23 people in the control group were survived by using endotracheal intubation again. (Table 1)

**Table 1:** Some medical indices in study and control groups

Groups	Cases	Time of invasive MV	Time of non-invasive MV	Time of total MV	Number of VAP	Patients died in hospital	Re-intubation rate
	(n)	(d)	(d)	(d)	(n)	(%)	(%)
Study group	38	8.56 ±047	5.50 ±0.61	12.23 ±0.40	5	7 (21.78%)	3(7.9%)
Control group	56	12.45±1.18	12.5 ±1.18	18.50±0.61	23	25 (45.45%)	11 (19.60%)

## Discussion

Weaning from mechanical ventilation for COPD patients is a challenge in clinic for a long time, because of long course of diseases, recurrent cough and wheeze with infection in COPD patients, which lead to respiratory muscle fatigue. In addition, balance of normal flora was inevitably destroyed as a result of using antibiotics long term, and influenced the body’s digestion and absorption. Patients will in high emergency condition when exacerbations, and then muscular atrophy will happen, even if it is timely to add exogenous proteins, it can’t change organization protein consumption of their own, so mal-nutrition is common in most of COPD patients. For those patients who have weaning difficulties, they will get pulmonary infection aggravated even though infection is controlled, because of respiratory muscles fatigue and weakness, sputum drained difficultly, obstructed airways repeatedly.

It is well known that during long-term mechanical ventilation, due to implementation of an invasive artificial airway, ventilator associated pneumonia (VAP) requiring repeated treatment often occurs. The results show that occurring rate of VAP in the study group is obviously lower than those of the control group (P<0.01). The decrease of the incidence rate of VAP can improve the effect of treatment and decrease the medical expenditure. In a way, increasing BMI can reduce dangerous factors of VAP, strengthen the body’s immunity and decrease mortality risk. The results show that 23 people in the control group (BMI<21, but their BMI>21 by enteral nutrition combined with parenteral nutrition in treatment) were survived by using endotracheal intubation again. In this way, we know BMI is important.

Pulmonary infection control window (PIC window) will appear early if the antibiotic is administrated reasonably, although the treatment of COPD is a comprehensive process. There is no significant difference between these two groups on analysis of invasive mechanical ventilation time, and infection could be controlled rapidly during the short period of invasive mechanical ventilation. These COPD cases require invasive mechanical ventilation again as a result of respiratory muscle fatigue and sputum drained difficulty followed by non-invasive mechanical ventilation. Compared with the control group, the study group had shorter duration of invasive mechanical ventilation, non-invasive mechanical ventilation time, total mechanical ventilation time, in re-intubation rate (P<0.01). Body mass index (BMI) is recommended as an important marker to reflect total nutrition of body. Capability of respiratory muscle and sputum drained of the COPD patients can be improved by strengthening nutrition, and the time of mechanical ventilation can be shorten. Therefore, BMI as an important reference index can be used to estimate the weaning from sequential mechanical ventilation for severity COPD patients.

The results show that weaning successfully from mechanical ventilation is very impossible when the BMI<21. In one of the studies by A Anzueto et al[5], there were no differences in the patients’ outcomes (duration of mechanical ventilation, duration of weaning, length of stay in the intensive care unit, length of stay in the hospital and mortality in the intensive care unit and in the hospital) based on body mass index categories, but they found a similar trend although they did not find statistically significant differences that body mass index was associated with risk-adjusted hospital mortality among mechanically ventilated adults with acute lung injury. Lower body mass index was associated with higher odds of death, whereas overweight and obese were associated with lower odds[5]. It is superficial to use BMI as an important reference index which estimates the weaning from sequential mechanical ventilation for severity COPD patients, but as one effective reference index. If using pulmonary infection control window (PIC window) as the switch point for transferring from invasive to noninvasive mechanical ventilation, BMI as a time point of weaning from sequential invasive-noninvasive mechanical ventilation need further clinical research.

## Declarations

This article has been published as part of *BMC Emergency Medicine* Volume 13 Supplement 1, 2013: Proceedings of the 2012 Emergency Medicine Annual Congress. The full contents of the supplement are available online at http://www.biomedcentral.com/bmcemergmed/supplements/13/S1.

The publication costs for this article was funded by PLA NO.155 Central Hospital.
